# Changes in Motivation, Socialization, Wellness and Mental Health in Youth Long-Distance Runners During COVID-19 Social Distancing Restrictions

**DOI:** 10.3389/fspor.2021.696264

**Published:** 2021-09-06

**Authors:** David M. Bazett-Jones, Micah C. Garcia, Jeffery A. Taylor-Haas, Jason T. Long, Mitchell J. Rauh, Mark V. Paterno, Kevin R. Ford

**Affiliations:** ^1^Motion Analysis and Integrative Neurophysiology Laboratory, School of Exercise and Rehabilitation Sciences, University of Toledo, Toledo, OH, United States; ^2^Division of Occupational Therapy and Physical Therapy, Cincinnati Children's Hospital Medical Center, Cincinnati, OH, United States; ^3^Motion Analysis Laboratory, Division of Occupational and Physical Therapy, Cincinnati Children's Hospital Medical Center, Cincinnati, OH, United States; ^4^Doctor of Physical Therapy Program, San Diego State University, San Diego, CA, United States; ^5^Division of Sports Medicine, Cincinnati Children's Hospital Medical Center, Cincinnati, OH, United States; ^6^College of Medicine, University of Cincinnati, Cincinnati, OH, United States; ^7^Department of Physical Therapy, Congdon School of Health Sciences, High Point University, High Point, NC, United States

**Keywords:** anxiety, enjoyment, nutrition, sleep, cross-country, track and field, adolescent

## Abstract

**Purpose:** The COVID-19 pandemic impacted the sporting and exercise activities of millions of youth. Running is an activity that could be maintained while social distancing restrictions were implemented during the pandemic. However, a recent study has indicated that youth runners reported lower running distance, frequency, and intensity during COVID-19. The reason for this reduction and the impact on overall well-being is unknown. Therefore, the purpose of this study was to determine if the social distancing restrictions during the 2020 COVID-19 pandemic influenced running motives, socialization, wellness and mental health in youth long-distance runners.

**Methods:** A customized, open online questionnaire was provided to runners 9–19 years of age who participated in long-distance running activities including team/club cross-country, track and field (distances ≥800 m), road races, or recreational running. Participants responded to questions about demographics, motive for running, and wellness (sleep quality, anxiety, running enjoyment, food consumption quality) 6-months before as well as during social distancing restrictions due to COVID-19. Wilcoxon signed rank tests compared differences for ratio data and Chi-square tests were used to compare proportions before and during COVID-19 social distancing restrictions. Statistical significance was set at *p* ≤ 0.05.

**Results:** A total of 287 youth long-distance runners (male = 124, female = 162, unspecified = 1; age = 15.3 ± 1.7 years; running experience = 5.0 ± 2.3 years) participated. Compared to their pre-COVID-19 responses, youth long-distance runners reported lower overall motivation to run (*p* < 0.001) and changes to most motive rankings (*p* < 0.001 to *p* = 0.71). The proportion of youth running alone increased during COVID-19 (65.8%) compared to pre-COVID-19 (13.8%, *p* < 0.001). Youth long-distance runners also reported less running enjoyment (*p* = 0.001), longer sleep duration (*p* < 0.001), lower sleep quality (*p* = 0.05), more anxiety (*p* = 0.043), and lower food quality consumed (*p* < 0.001) during COVID-19 social distance restrictions.

**Conclusion:** The COVID-19 social distancing restrictions resulted in significant decreases in motivation and enjoyment of running. The removal of competition and team-based interactions likely had a role in these decreases for this population. Continuing team-based activities (e.g., virtual) during social distancing may help with maintaining motivation of youth long-distance runners. Reduced running occurred concurrently with reduced overall well-being of youth long-distance runners during the COVID-19 pandemic.

## Introduction

The World Health Organization (WHO) declared Coronavirus Disease 2019 (COVID-19) a pandemic on March 11th, 2020 (World Health Organization, 2020). As a result, schools across the United States canceled in-person classes and extracurricular activities such as interscholastic sports. Millions of youth who were typically physically active through sports were suddenly required to stay at home. The health of adolescent athletes has been reported to be negatively impacted by the cancelation of sport seasons during the pandemic (McGuine et al., 2020).

The health of adolescent athletes who participate in individual sports like cross-country may not have experienced the same negative experiences during COVID-19 as team sports athletes (McGuine et al., 2020). That is because running can be performed outside of facilities and with the appropriate social distancing required during COVID-19. However, during spring 2020 of the COVID-19 pandemic, youth long-distance runners reported a decrease in their running habits, including reduced running distance (decreased 14.0%), total number of runs per week (decreased 14.0%), and number of hard runs per week (decreased 27.0%) (Bazett-Jones et al., 2020). This is in contrast with adult runners that reportedly increased their running behaviors following social distancing restrictions (DeJong et al., 2021; Holmes et al., 2021; Mosqueira-Ourens et al., 2021). Adults who made more changes to their running behaviors were injured more but these changes were due to multiple factors (Holmes et al., 2021). While changes in running habits have been described, motives to run may provide a greater depth of understanding why running habits increased or decreased during COVID-19 social distancing.

Motivation is important for maintaining physical activity and sport. Youth often choose to participate in sports based on social and competition motivational factors (Verkooijen et al., 2009). Adult and youth long-distance runners have reported that socialization is a strong motive to run (Springer, 2013). As socialization has been negatively impacted during COVID-19 worldwide (Ammar et al., 2020b), it is reasonable to assume that socialization has been impacted in runners. This assumption was supported as adult runners who demonstrated a decline in motivation and transition from competition and socialization motives to fitness, occupying time, and stress relief motives (DeJong et al., 2021). While less has been published in youth runners, competition may also be an important factor related to motivation and enjoyment in children (Chalkley et al., 2020). As sports seasons and competitions were canceled due to COVID-19, youth runners may have become less motivated to run and felt less enjoyment, potentially contributing to the reported reductions in running (Bazett-Jones et al., 2020). Understanding why youth long-distance runners change their running habits provides greater insights that could help coaches and health care professionals prepare youth runners return to running following a period of reduced running.

In addition to reducing physical activity, the COVID-19 pandemic has contributed to worsening overall wellness across the globe. Sleep problems have been reported as a result of the COVID-19 pandemic (Voitsidis et al., 2020; Abid et al., 2021; Trabelsi et al., 2021). During COVID-19 confinement, consumption of more unhealthy foods increased and more snacking occurred (Ammar et al., 2020a; Di Renzo et al., 2020; Papandreou et al., 2020; Sanchez-Sanchez et al., 2020). Elevated stress and anxiety levels have consistently been reported during COVID-19 social distancing and isolation (Di Renzo et al., 2020; Lopez-Bueno et al., 2020b; Papandreou et al., 2020). These findings of increased stress and anxiety have also been observed in youth athletes; however, most of these athletes participate in sports that cannot be competed in or practiced during social distancing (McGuine et al., 2020, 2021). Since many runners can continue their physical activity without access to facilities, they may be at a lower risk of worsening mental health during COVID-19 social distancing. Recently, Bazett-Jones et al. (2020) found that youth runners did not maintain physical activity and reported reduced running habits during COVID-19. Therefore, it appears that youth runners may not have benefitted as expected from the potential mental health effects of physical activity during COVID-19. Thus, it is important to understand the impact of COVID-19 social distancing on the mental health and overall wellness of youth runners.

The purpose of this study was to determine if the social distancing restrictions during the 2020 COVID-19 pandemic influenced running motivation, socialization, wellness and mental health in youth long-distance runners. We hypothesized that (1) self-reported motivation levels for running would decrease, (2) competition-related motives would be less motivating, (3) social distancing restrictions would result in decreased group running behaviors and increased running alone, (4) sleep quantity and quality would decrease, (5) quality of food intake would decrease, and (6) anxiety level would increase during COVID-19.

## Materials and Methods

### Participants

Youth long-distance runners were recruited from middle- and high-schools across the United States. Recruitment emails were sent to athletic directors or coaches asking them to provide the recruitment materials to the runners on their cross-county team as well as to encourage the runners to participate in the study. Participants from previous running research studies who qualified for this study were also recruited. Information about the study and survey links were sent between May and June 2020, with a reminder invitation sent 10–14 days after the initial invitation. The survey link was also shared on various social media platforms and others were encouraged to share the survey. Participants were included in the study if they were 9–19 years of age and participated in long-distance running activities including team/club cross-country, track and field (distances ≥800 m), road races, or recreational running. Participants were excluded if their primary sport was not cross-country or track and field and they did not participate in long-distance running activities and/or if they resided outside of the United States. Study procedures were approved by the Institutional Review Boards at the University of Toledo and Cincinnati Children's Hospital Medical Center. Consent was obtained for participants 18 or 19 years of age and parental permission/child assent were obtained for participants 9–17 years old. Parents were encouraged to assist their child when necessary. The participants did not receive incentives for participating.

### Questionnaire

A customized, open online questionnaire was provided to the participants through password-protected instruments from Qualtrics (SAP SE, Germany) or Research Electronic Data Capture (REDCap, Vanderbilt University, TN). The Checklist for Reporting Results of Internet E-Surveys (CHERRIES) was used to ensure study quality (Eysenbach, 2004). Both instruments and institutions used identical questions. The questionnaire required ~10 min to complete. It consisted of 83 items that were separated among a minimum of 13 screens. The questions inquired about the participant's demographics (age, sex, state, years of running experience, recreational/competitive runner), running habits, running-related injuries (RRIs), running motivation, and wellness (sleep quality, anxiety, running enjoyment, food consumption quality) 6-months before as well as during social distancing restrictions due to COVID-19 (Bazett-Jones et al., 2020; DeJong et al., 2021). Questions were presented in a consistent order with adaptive questioning dependent on participant responses. Participants were asked to complete the survey with no time restrictions. Participants had the opportunity to review and change their responses prior to submitting them. Once the submission was made, no changes to their responses were allowed.

Running motivation questions included a 5-point Likert scale for how motivated the participant felt to run on a typical day (extremely motivated, somewhat motivated, no more/no less motivated, somewhat not motivated, extremely not motivated). Participants also ranked six motives (competition/races, socialization, enjoyment/pleasure, occupy free time, stress relief, exercise/fitness) for running from most motivating to least motivating. These motives categories have been used previously in research with adult runners during COVID-19 (DeJong et al., 2021). While other questionnaires on running motivation exist [e.g., Motivation of Marathoners Scale (Zach et al., 2017)], we agreed with other authors on its validity in a youth population (Malchrowicz-Mosko et al., 2020) who were non-marathoners. Runners were also asked about their most common running companion(s) [“by myself,” “with my family member(s),” “with my friend(s),” “with my teammate(s),” “with my coach(es)”].

Sleep duration was self-reported in response to the question, “how many hours of sleep per night were you sleeping?” relative to either before or during social distancing restrictions. Children and adolescents have been reported to accurately report sleep duration compared to quantitative measures (Combs et al., 2019). Sleep quality was self-reported using a 4-point Likert scale describing sleep quality (very bad, fairly bad, fairly good, very good) from the Pittsburg Sleep Quality Index (Buysse et al., 1989). Similar Likert scales have been used previously to assess sleep quality in athletes (Andrade et al., 2016, 2019; Brandt et al., 2017). Participants were grouped as reporting good sleep quality (fairly good, very good) or poor sleep quality (fairly bad, very bad).

Anxiety of youth long-distance runners was measured using a 100-point visual analog scale (anchored as “very anxious” [0] to “very calm” [100]). Anxiety was defined as feelings of worry, nervousness, or unease about future events. Visual analog scales have been reported to be capable of validly measuring anxiety (Williams et al., 2010).

Enjoyment of running was assessed with a visual analog scale (anchored by “no enjoyment” [0] to “complete enjoyment” [100]) from previous studies measuring running enjoyment in adult runners (Carnes et al., 2015, 2016).

Youth long-distance runners were asked, “How would you describe the quality of foods that you ate regularly?” relative to either before or during social distancing restrictions. Their self-perception of quality of foods that they regularly consume was measured with a visual analog scale (anchored by “not healthy” [0] and “healthy” [100]).

Incomplete responses were excluded and completed surveys were used for analysis. The IP address was recorded for each response. If duplicate IP addresses were identified, the responses were checked for originality and responses from the same IP address were only used if the responses were clearly different (e.g., two siblings using the same home computer, or two teammates using a common school computer).

### Statistical Analyses

Data analysis was conducted using SPSS statistical software (version 26, IBM Inc., Armonk, NY). Data were found non-normally distributed (Kolmogorov-Smirnov, *p* ≤ 0.05). Wilcoxon signed rank tests compared differences before and during COVID-19 social distancing restrictions for motivation to run on a typical day, rank of running motives, sleep duration, anxiety, enjoyment of running, and quality of food consumed. Chi-square tests compared the proportions of participants who reported poor sleep quality and of running companions before and during COVID-19 social distancing restrictions. For the chi-square tests, adjusted standardized residuals were used for pairwise comparisons with significant differences occurring when *z* ≥ 1.96. Statistical significance was set at *p* ≤ 0.05.

## Results

Of the 576 survey attempts, 287 participants (male = 124, female = 162, unspecified = 1; age = 15.3 ± 1.7 years; running experience = 5.0 ± 2.3 years) met inclusion criteria and were fully completed (completion rate = 49.8%). The most common reason for exclusion was due to being outside the required age range. In total, 235 (81.9%) and 52 (18.1%) participants reported they were competitive and recreational runners, respectively. Participants resided in 20 different states with most from the Midwest of the United States (Bazett-Jones et al., 2020).

Compared to their pre-COVID-19 responses, youth long-distance runners reported lower overall motivation to run (*p* < 0.001) during COVID-19 social distance restrictions (Table 1). The motives for running were also significantly changed. Compared to their pre-COVID-19 responses, youth long-distance runners reported increases in the average motive rankings of running to occupy free time (*p* < 0.001), relieve stress (*p* < 0.001), and for exercise/fitness benefits (*p* < 0.001) during COVID-19 social distancing restrictions. Running for competition (*p* < 0.001) and socialization (*p* < 0.001) were reported as having lower average motive rankings during COVID-19 social distancing restrictions compared to pre-COVID-19. No difference was observed in the motive ranking of running for pleasure (*p* = 0.71) between pre- and during COVID-19 social distancing restrictions. Pre-COVID-19, the most commonly reported greatest motive (ranked first) to run was competition (*n* = 151, 58.1%; Table 2), followed by exercise/fitness benefits (*n* = 51, 19.6%) and socialization (*n* = 31, 11.9%). Following COVID-19 social distancing restrictions, the most commonly reported greatest motive (ranked first) to run was exercise/fitness benefits (*n* = 107, 41.2%) followed by competition (*n* = 60, 23.1%) and to occupy free time (*n* = 33, 12.7%).

**Table 1:** Differences in running enjoyment, sleep habits, feelings of anxiety, food quality, and running motivation pre- and during COVID-19 social distancing restrictions.

**Variable**	**Median [IQR]**		**Mean ± standard deviation**		***P***
	**Pre-COVID-19**	**During COVID-19**	**Pre-COVID-19**	**During COVID-19**	
Running enjoyment^‡^	76.0 [54.0, 92.0]	65.0 [46.0, 83.8]	68.4 ± 29.5	62.4 ± 27.0	0.001^*^
Sleep duration [hrs]	8.0 [7.0, 8.0]	8.0 [7.8, 9.0]	7.6 ± 1.2	8.3 ± 1.5	<0.001^*^
Feelings of anxiety^&^	63.0 [38.0, 82.0]	52.5 [30.3, 82.8]	59.3 ± 27.1	55.8 ± 28.6	0.04^*^
Food quality^‡^	66.5 [51.0, 83.0]	61.0 [45.0, 80.0]	65.6 ± 21.9	60.1 ± 25.5	<0.001^*^
Motivation^†^	2.0 [1.0, 2.0]	2.0 [2.0, 4.0]	1.9 ± 0.7	2.7 ± 1.3	<0.001^*^

† *1, extremely motivated; 5, extremely not motivated;*

‡ *0, no enjoyment/not health; 100, complete enjoyment/healthy;*

& *0, very anxious; 100, very calm*.

* *Statistical significance (p ≤ 0.05)*.

**Table 2:** Differences in motivation factors pre- and during COVID-19 social distancing restrictions.

**Variable**	**Median [IQR]**		**Mean ± standard deviation**		***P***
	**Pre-COVID-19**	**During COVID-19**	**Pre-COVID-19**	**During COVID-19**	
Competition	1.0 [1.0, 3.0]	4.0 [2.0, 5.0]	2.2 ± 1.7	3.8 ± 1.9	<0.001^*^
Socialization	3.0 [2.0, 4.0]	5.0 [4.0, 6.0]	3.3 ± 1.6	4.7 ± 1.6	<0.001^*^
Enjoyment/pleasure	3.0 [3.0, 4.0]	4.0 [2.3, 4.0]	3.4 ± 1.3	3.5 ± 1.3	0.71
Free time	6.0 [5.0, 6.0]	3.0 [2.0, 4.0]	5.1 ± 1.1	3.2 ± 1.4	<0.001^*^
Stress relief	4.0 [3.0, 5.0]	4.0 [2.3, 5.0]	4.2 ± 1.4	3.6 ± 1.5	<0.001^*^
Exercise	2.5 [2.0, 4.0]	2.0 [1.0, 3.0]	2.7 ± 1.4	2.2 ± 1.4	<0.001^*^

*1, greatest motivator; 6, lowest motivator*.

* *Statistical significance (p ≤ 0.05)*.

During COVID-19 social distancing restrictions, a greater proportion of youth long-distance runners reported mostly running alone (pre-COVID-19 *n* = 36, 13.8%; during COVID-19 *n* = 171, 65.8%; *z* = 12.1; Table 3) and with family (pre-COVID-19 *n* = 8, 3.1%; during COVID-19 *n* = 41, 15.8%; *z* = 5.0) while a lesser proportion reported mostly running with teammates (pre-COVID-19 *n* = 196, 75.4%; during COVID-19 *n* = 24, 9.2%; *z* = −15.3). Similar proportions were noted observed for running with coaches (pre-COVID-19 *n* = 4, 1.5%; during COVID-19 *n* = 1, 0.4%; *z* = −1.3) and friends (pre-COVID-19 *n* = 16, 6.2%; post-COVID-19 *n* = 23, 8.8%; *z* = 1.2).

**Table 3:** Proportions of most common running socialization pre- and during COVID-19 social distancing restrictions reported as n (%).

	**Pre-COVID-19**	**During COVID-19**	**Adjusted residual [z]**
Alone	36 (13.8)	171 (65.8)	12.1^*^
With teammates	196 (75.4)	24 (9.2)	−15.3^*^
With coaches	4 (1.5)	1 (0.4)	−1.3
With friends	16 (6.2)	23 (8.8)	1.2
With family	8 (3.1)	41 (15.8)	5.0^*^

* *Statistical significance (z ≥ 1.96)*.

**Figure 1:**
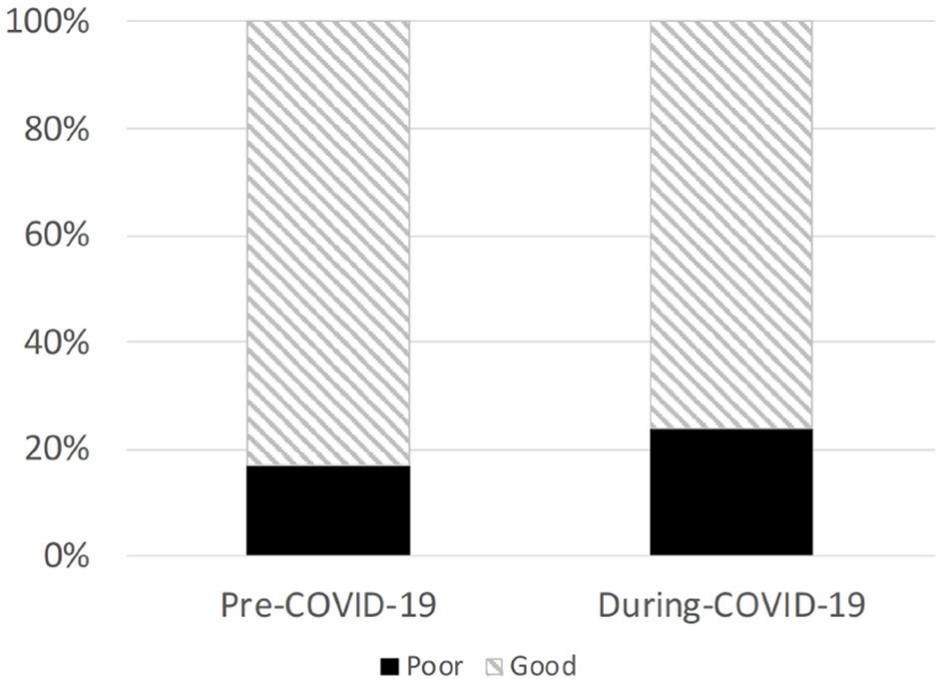
Sleep quality pre- and during-COVID-19 social distancing restrictions. A significantly greater proportion of participants reported poor sleep quality during-COVID-19 compared to pre-COVID-19 (*p* = 0.05).

Compared to their pre-COVID-19 responses, youth long-distance runners reported less enjoyment running (*p* = 0.001), longer sleep duration (*p* < 0.001), more anxiety (*p* = 0.04), and lower food quality consumed (*p* < 0.001) during COVID-19 social distance restrictions (Table 1). A higher proportion of youth long-distance runners reported poor sleep quality during COVID-19 social distancing restrictions than pre-COVID-19 (pre-COVD-19 *n* = 44, 41.5%; during COVID-19 *n* = 62, 58.5%; *p* = 0.05) (Figure 1).

## Discussion

The purpose of this study was to determine if there were changes to running motivation, socialization, wellness and mental health in youth long-distance runners due to social distancing restrictions during the 2020 COVID-19 pandemic. We found that youth long-distance runners' motivation, motives, sleep duration and quality, quality of food consumed, running socialization, and anxiety were mostly negatively impacted by COVID-19 social distancing restrictions. These changes are consistent with other reported findings in youth athletes (McGuine et al., 2020, 2021) and could have implications for the health and well-being of youth long-distance runners, which requires further investigation.

Youth long-distance runners' motivation and motives for running were different during COVID-19, likely due to the elimination of competitive seasons or events. Overall, self-reported motivation was significantly less during COVID-19 compared to pre-COVID-19 (8.8% decrease). The top ranked motive, on average, for youth long distance runners pre-COVID-19 was competition but this motive fell to 5^th^ highest ranked motivator during COVID-19, which was a significant difference. Most youth runners ranked competition as their number one motivator pre-COVID-19 but this decreased to less than a quarter during COVID-19. Competition motivates youth to run (Chalkley et al., 2020) and removal of this competition motivator likely contributed to the decrease in running seen in this population during the pandemic (Bazett-Jones et al., 2020). The only other motivator to decrease in its average ranking was socialization, moving from the 3^rd^ highest ranked motivator, on average, pre-COVID-19 to the lowest ranked motivator during COVID-19. Cancellation of these competitive seasons also impacted youth long-distance runner socialization in that practice with teammates was no longer possible, which is also a strong motivator in runners (Springer, 2013; Chalkley et al., 2020). These results are consistent with changes in motivators in adult runners during the pandemic. DeJong et al. (2021) reported decreases in competition and socialization motivators in adult runners during COVID-19 than reported prior to COVID-19. The change in motivators for youth long-distance runners may also have influenced the significant decrease in self-reported enjoyment of running during COVID-19 social distancing restrictions compared to pre-COVID-19. Due to the removal of competition and social interactions of the team, it may be that the youth long-distance runner in our study experienced less enjoyment due to the lack of these extrinsic motivators.

The reduction in socialization as a motivator was also confirmed in our study as significant changes were noted in the proportions of running companions during COVID-19 compared to pre-COVID-19. Pre-COVID-19 restrictions, running took place with other people 86.2% of the time, but this fell to 34.2% during COVID-19. Cancellation of competitive seasons and team practices significantly reduced the opportunity for socialization with teammates. Almost five times as many runners ran alone during COVID-19 (*n* = 171/248) compared to pre-COVID-19 (*n* = 31/248) social distancing restrictions. This significant increase in running alone could be related to the lower enjoyment or motivation reported. Collegiate runners have reported greater enjoyment while running with others than alone (Carnes et al., 2015). Enjoyment during sport participation is an important factor to maintaining involvement and reducing dropout (Gardner et al., 2017). Maintaining enjoyment in this population during short- or long-term isolation (i.e., COVID-19, injury) is likely important to the youth long-distance runner. Similar isolation may be experienced in runners removed from competition due to injury. Strategies for maintaining socialization and enjoyment should be important topics of future investigations in this population. Parental involvement has been reported to positively influence enjoyment in youth tennis players (Hoyle and Leff, 1997). While our findings indicated an increase of runners running with their family members, the percentage doing so was fairly low so we are not certain if it may have offset the reduction in enjoyment.

As competition and socialization motivators and environments changed, running motivators of exercise, stress relief, and occupying free-time all increased in their average ranking during COVID-19 compared to pre-COVID-19. Interestingly, running as an activity to occupy free time was ranked last pre-COVID-19 (0% ranked as #1) but became the 2^nd^ highest ranked motivator during COVID-19 (12.7% ranked as #1). In adult runners, an increase in motivation to occupy free time has also been reported (DeJong et al., 2021). In the same study (DeJong et al., 2021), however, the adult runners did not show increases in other motivators. While youth long-distance runners reduced their running frequency and distance during COVID-19 (Bazett-Jones et al., 2020), the health benefits (exercise, stress relief) of running stayed as a strong motivator. Running for exercise was ranked #1 by almost 20% of participants' pre-COVID-19 and this increased to over 40% during COVID-19. These findings demonstrate that many youth long-distance runners are motivated to continue running despite the lack of competition and socialization. Further research on subgroups of those who are motivated by different factors should be undertaken to investigate their long-term impact on physical activity once competition is removed due to matriculation.

Developing and maintaining intrinsic motivators (i.e., exercise, stress relief) is important for youth athletes (Bergeron et al., 2015) for many different reasons, including maintaining mental health (Dore et al., 2016). The global pandemic of COVID-19 has had a negative impact on mental health. During COVID-19 social distancing restrictions, increased anxiety (Di Renzo et al., 2020; Papandreou et al., 2020) and decreased overall mental health (Liang et al., 2020) have been reported in the general adolescent and youth populations. Our findings are consistent with these studies (Di Renzo et al., 2020; Liang et al., 2020; Papandreou et al., 2020) in that the youth long-distance runners reported a significant increase in anxiety during COVID-19 compared to their self-reported pre-COVID-19 levels. This elevation in anxiety could be related to a decrease in running in this population (Bazett-Jones et al., 2020). Abruptly stopping running for as little as 2 weeks has been reported to cause an increase in depressive symptoms in regular runners (Morris et al., 1990). This is important because multiple studies in adults have reported that adherence to physical activity guidelines has been shown to mitigate increases in anxiety during COVID-19 social distancing restrictions (Jacob et al., 2020; Lopez-Bueno et al., 2020a). Further, it is unknown if there are long-term ramifications from the reduced mental wellness.

In our study, youth long-distance runners reported positive and negative changes to their wellness habits. They reported an increase in sleep duration but a higher proportion reported poor sleep quality during COVID-19 social distancing restrictions. Increased sleep duration during COVID-19 confinement has been consistently reported in adolescents (Kaditis et al., 2021; Ramos Socarras et al., 2021). However, long-distance runners have been reported to have inadequate sleep compared to recommendations (Garcia et al., 2021). Increase in sleep duration is desirable because increased sleep duration improves attention, behavior, learning, memory, emotional regulation, quality of life, and mental and physical health (Paruthi et al., 2016; Fox et al., 2020). Symptoms of anxiety, stress, and depression are more likely to be reported in adolescent athletes with poor sleep quality (Gomes et al., 2017; Potter et al., 2020). Unfortunately, we are unable to discern if poor sleep quality is a result of increased anxiety or if increased anxiety is a result of poor sleep quality. It is unknown what long-term implications these changes may have, including how sleep quality and anxiety interact.

Self-reported food quality significantly decreased during COVID-19 restrictions; however, this should be interpreted with caution do to the subjective, single-item nature of this question. Our limited results are in agreement with published literature demonstrating worsening food quality and poor nutritional habits in youth and adults during COVID-19 (Ammar et al., 2020a; Di Renzo et al., 2020; Lopez-Bueno et al., 2020b; Papandreou et al., 2020). Consumption of unhealthy food, increased snacking, increased overall number of meals, and/or eating out of control may all contribute to a lower quality diet (Ammar et al., 2020a). This worsening of consumed food quality could be attributed to reduced motivation, eating in response to boredom or anxiety or other mood-driven eating (Di Renzo et al., 2020; Papandreou et al., 2020). To our knowledge, the nutritional habits of youth long-distance runners has not been reported during COVID-19 and therefore, we are unable to understand the extent and meaningfulness of this reduction in self-reported food quality in our study sample.

Several limitations in this study are noteworthy. First, the cross-sectional nature of an online survey may have introduced some recall bias which influenced the reporting of some variables of interest. We attempted to minimize the impact of this by limiting the recall period to 6 months prior to COVID-19. Social distancing restrictions also differed in extent and timeline based on state government decisions, likely leading to variability in the stay-at-home orders affecting each participant. Second, our study was also limited in its measurement at one time point. Future research should study these variables in runners during the season following the COVID-19 restrictions to provide evidence of a long-lasting impact on youth long-distance runners. Third, though we attempted to get a broad sense of the COVID-19 impact on the runners, the responses were weighted more heavily among a few US states which may have introduced a geographic bias. Finally, the questions and/or scales used in this study have received limited investigation into their validity and reliability. The questions/scales may be missing or unable to measure important components of various concepts regarding motives, sleep, food quality, and anxiety. Further research is needed on these various topics in adolescents and long-distance runners.

Within the context of these limitations, this study adds to the current literature regarding the negative effects that large-scale cancellation of a sports had on youth long-distance runners during the COVID-19 pandemic (McGuine et al., 2020, 2021). Youth long-distance runners experienced changes in motivation, lower enjoyment of running, changes in wellness, and more anxiety even though they were not necessarily restricted in their ability to run, only the context and reasons for which they participated. However, running distance and frequency were reduced (Bazett-Jones et al., 2020), which may have been influenced by the removal of competition and social motivators. The use of technology to create competition or increase social interactions during times of increased isolation may be one solution to these barriers that warrants future investigation.

## Data Availability Statement

The raw data supporting the conclusions of this article will be made available by the authors, without undue reservation.

## Ethics Statement

The studies involving human participants were reviewed and approved by University of Toledo Institutional Review Board. Written informed consent to participate in this study was provided by the participants' legal guardian/next of kin.

## Author Contributions

All authors listed have made a substantial, direct and intellectual contribution to the work, and approved it for publication.

## Conflict of Interest

The authors declare that the research was conducted in the absence of any commercial or financial relationships that could be construed as a potential conflict of interest.

## Publisher's Note

All claims expressed in this article are solely those of the authors and do not necessarily represent those of their affiliated organizations, or those of the publisher, the editors and the reviewers. Any product that may be evaluated in this article, or claim that may be made by its manufacturer, is not guaranteed or endorsed by the publisher.
